# Urinary Concentrations of Bisphenol A and 4-Nonylphenol in a Human Reference Population

**DOI:** 10.1289/ehp.7534

**Published:** 2004-12-20

**Authors:** Antonia M. Calafat, Zsuzsanna Kuklenyik, John A. Reidy, Samuel P. Caudill, John Ekong, Larry L. Needham

**Affiliations:** Division of Laboratory Sciences, National Center for Environmental Health, Centers for Disease Control and Prevention, Atlanta, Georgia, USA

**Keywords:** bisphenol A, exposure, human, NHANES III, nonylphenol, urine

## Abstract

Bisphenol A (BPA) is used to manufacture polycarbonate plastic and epoxy resins, which are used in baby bottles, as protective coatings on food containers, and for composites and sealants in dentistry. 4-Nonylphenol (NP) is used to make nonylphenol ethoxylates, nonionic surfactants applied as emulsifying, wetting, dispersing, or stabilizing agents in industrial, agricultural, and domestic consumer products. The potential for human exposure to BPA and NP is high because of their widespread use. We measured BPA and NP in archived urine samples from a reference population of 394 adults in the United States using isotope-dilution gas chromatography/mass spectrometry. The concentration ranges of BPA and NP were similar to those observed in other human populations. BPA was detected in 95% of the samples examined at concentrations ≥0.1 μg/L urine; the geometric mean and median concentrations were 1.33 μg/L (1.36 μg/g creatinine) and 1.28 μg/L (1.32 μg/g creatinine), respectively; the 95th percentile concentration was 5.18 μg/L (7.95 μg/g creatinine). NP was detected in 51% of the samples examined ≥0.1 μg/L. The median and 95th percentile concentrations were < 0.1 μg/L and 1.57 μg/L (1.39 μg/g creatinine), respectively. The frequent detection of BPA suggests widespread exposure to this compound in residents of the United States. The lower frequency of detection of NP than of BPA could be explained by a lower exposure of humans to NP, by different pharmacokinetic factors (i.e., absorption, distribution, metabolism, elimination), by the fact that 4-*n*-nonylphenol—the measured NP isomer—represents a small percentage of the NP used in commercial mixtures, or a combination of all of the above. Additional research is needed to determine the best urinary biomarker(s) to assess exposure to NP. Despite the sample population’s nonrepresentativeness of the U.S. population (although sample weights were used to improve the extent to which the results represent the U.S. population) and relatively small size, this study provides the first reference range of human internal dose levels of BPA and NP in a demographically diverse human population.

In developed countries, humans are potentially exposed to a wide range of chemicals present in commonly used products. For the most part, no information exists about the extent of the human exposure to these chemicals, and the potential toxic effects of these compounds are largely unknown. Bisphenol A (2,2′-bis[4-hydroxyphenyl]propane; BPA) and alkylphenols (APs) are among these chemicals. BPA is used to manufacture polycarbonate plastic and epoxy resins, which are used in baby bottles, as protective coatings on food containers, and for composites and sealants in dentistry (Arenholt-Bindslev et al. 1999; Howe et al. 1998; Sajiki and Yonekubo 2003). APs are used to make alkylphenol ethoxylates (APEs), widely used nonionic surfactants. APEs are applied as emulsifying, wetting, dispersing, or stabilizing agents in numerous industrial, agricultural, and domestic consumer products including detergents and pesticide formulations (Montgomery-Brown and Reinhard 2003; Ying et al. 2002). 4-Nonylphenol (NP) is one of the most widely used and studied APs [European Commission (EC) 2002a; U.S. Environmental Protection Agency (EPA) 2003]. BPA and NP can be released into the environment during the manufacturing process and by leaching from the final products (Guenther et al. 2002; Maguire 1999; Staples et al. 1998). Because of the widespread use of BPA and NP, the potential for human exposure is high.

Toxicologic studies of laboratory animals suggest that exposure to BPA and NP is associated with morphologic, functional, and behavioral anomalies related to reproduction. NP is estrogenic *in vitro* and *in vivo* (Kwack et al. 2002), interferes with the estrous cycle and pubertal onset in rats (Kim et al. 2002; Laws et al. 2000), and shows aquatic toxicity at low (micrograms per liter) concentrations (Hemmer et al. 2001; Ying et al. 2002). Exposure of rodent fetuses to low BPA doses of 20–400 μg/kg/day produces postnatal estrogenic effects, including reduced daily sperm production and increased prostate gland weight in males, alteration in the development and tissue organization of the mammary gland, disruption of sexual differentiation in the brain, long-term deleterious effects in the vagina, and accelerated growth and puberty in females (Howdeshell et al. 1999; Kubo et al. 2003; Markey et al. 2001; Nagel et al. 1997; Schonfelder et al. 2002; vom Saal et al. 1998; Welshons et al. 1998; White et al. 1994). In mice treated with BPA at oral doses comparable with environmental exposure levels (20–100 μg/kg body weight/day), BPA appears to be a potent disruptor of meiosis, the cell division process that creates sperm or eggs, leading to aneuploidy (Hunt et al. 2003). Aneuploidy (an error in cell division) during meiosis is thought to be the most common known cause of mental retardation as well as being the leading genetic cause of pregnancy loss in humans (Hassold and Hunt 2001).

Data on human exposure to NP are scarce (Inoue et al. 2003; Kawaguchi et al. 2004). NP was measured in the urine samples of 10 healthy volunteers using column-switching liquid chromatography/mass spectrometry (Inoue et al. 2003). NP was detected at concentrations higher than the limit of detection (LOD; 0.3 ng/mL) in one sample. Recently, NP was measured in five urine and three plasma samples from eight adult volunteers by using stir-bar sorptive extraction-thermal desorption–gas chromatography/mass spectrometry (GC/MS) (Kawaguchi et al. 2004). The NP concentrations in urine were below the LOD (0.2 ng/mL).

The urinary BPA levels in various populations in Southeast Asia have been reported (Arakawa et al. 2004; Matsumoto et al. 2003; Ouchi and Watanabe 2002; Yang et al. 2003). BPA was measured in morning spot urine samples from two different groups of university students in Japan in 1992 (*n* = 50, 92% male) and 1999 (*n* = 56, 87.5% male). Most BPA was found in the urine as the glucuronide conjugate. The median urinary BPA levels in 1992 were approximately 2.2-fold higher than in 1999 (Matsumoto et al. 2003). In another study, BPA glucuronide was detected in all of the urine samples collected from 48 female Japanese college students at concentrations ranging from 0.2 to 19.1 ng/mL, with a median level of 1.2 μg/L (0.77 μg/g creatinine) (Ouchi and Watanabe 2002). The largest BPA nonoccupational exposure assessment reported urinary levels of BPA in a group of 73 adult Koreans (53.4% female). The geometric mean (GM) concentration of BPA was 9.54 μg/L (8.91 μg/g creatinine) (Yang et al. 2003). A recent study in Japan reported a median daily urinary excretion of BPA of 1.2 μg/day and a maximum daily intake of BPA per body weight to be 0.23 μg/kg/day based on the measurement of BPA in 24-hr urine samples collected from 36 men (Arakawa et al. 2004). This estimated maximum daily intake was lower than the temporary tolerable daily intake (10 μg/kg) set by the European Commission’s Scientific Committee in Food in 2002 (Arakawa et al. 2004; EC 2002b).

Occupational exposure to BPA has been assessed in a cross-sectional study involving 84 male workers in plastic plants in Japan (Hanaoka et al. 2002). Forty-two of the workers used bisphenol A diglycidyl ether (BADGE) and mixed organic solvents for spraying epoxy resin hardening agents, whereas the other 42 workers did not use BADGE. The median concentrations of urinary BPA were higher (*p* = 0.002) in the sprayers (1.06 μmol/mol creatinine) than in the nonexposed workers (0.52 μmol/mol creatinine). Similarly, the concentrations of urinary metabolites of the organic solvents used were significantly higher in the sprayers than in the nonexposed workers. In contrast, the median plasma concentrations of follicle-stimulating hormone (FSH) were lower (although within the normal range) in the sprayers than in the nonexposed workers (*p* = 0.022). The levels of FSH were slightly negatively correlated with those of BPA (correlation coefficient, −0.2, *p* = 0.071) but not with the metabolites of the organic solvents. On the basis of these results, the authors postulated that BPA may disrupt the secretion of gonadotropic hormones in men, although they also acknowledged that the clinical significance of these findings is still unclear and should be further investigated (Hanaoka et al. 2002).

Although no clear association has been established between human exposure to BPA or NP and adverse health effects, studies to investigate the prevalence of exposure to these environmental phenols are warranted because of their potential harmful effects. Here we report the levels of BPA and NP in urine from a demographically diverse sample of U.S. adults using a sensitive technique (Kuklenyik et al. 2003) as a tool for assessing the internal dose of these compounds.

## Materials and Methods

The urine samples analyzed for this study were selected from the Third National Health and Nutrition Examination Survey (NHANES III) callback cohort, a nonrepresentative subset of NHANES III composed of approximately 1,000 adults. The urine samples were all spot-urine samples, collected at different times throughout the day and were not necessarily first-morning voids. Creatinine adjustment was used to correct for urine dilution (Jackson 1966).

BPA and 4-*n*-nonylphenol (nNP), the linear chain NP isomer, were measured using a method based on an automated solid-phase extraction (SPE) coupled to isotope dilution-GC/MS (Kuklenyik et al. 2003). First, the urine samples were treated with β-glucuronidase to hydrolyze the glucuronide conjugates. Then, during the automated SPE process, BPA and nNP were both extracted from the deconjugated urine matrix and derivatized, using pentafluorobenzyl bromide, on commercial styrene-divinylbenzene copolymer-based SPE cartridges. After elution from the SPE column, the derivatized phenols in the SPE eluate were measured by isotope-dilution GC/MS. The limits of detection (LODs) for BPA and nNP in a 1-mL urine sample were 0.1 μg/L.

Quality control (QC) materials were analyzed along with the samples to assure the accuracy and reliability of the data. Low-concentration (QCL, 2–5 ng/mL) and high-concentration (QCH, 12–20 ng/mL) QC materials were prepared from a base urine pool—obtained from multiple anonymous donors as described previously (Kuklenyik et al. 2003)—dispensed in 5-mL aliquots and stored at −20°C. Each QC material was characterized by repeated measurements, spanned over at least 4 weeks, to define the mean concentrations and the 95% and 99% control limits of BPA and nNP. Each analytical run consisted of 40 (2 QCH, 2 QCL, 4 blanks, and 32 unknown) samples. The concentrations of the two QCH and the two QCL, averaged to obtain one measurement of QCH and QCL for each run, were evaluated using standard statistical probability rules.

The samples used for this study were stored securely at −70°C and may have been subject to repeated thaw/freeze cycles. Before analysis, the samples and QC materials were left to thaw overnight at 5°C. The concentrations of the analytes in the QCs remained essentially constant under these experimental conditions. Furthermore, QC materials reanalyzed after the initial characterization showed that BPA and nNP remained stable in the QC materials at −20°C for at least 1 year. Although the long-term stability of the analytes in the urine samples stored for > 1 year is not known, the QC data suggest that the integrity of the specimens is likely maintained and that chemical degradation of the phenols was undetectable.

To estimate total sample size, we used a standard formula *n* = *t*^2^*p*(1 − *p*)/*d*^2^, where *n* is the estimated sample size, *t* is the critical value associated with the desired statistical confidence level, and *d* is the maximum allowable error above or below the estimate of the true proportion (*p*) of the target population with measurable levels of the analyte(s) of interest (Peavy 1996). Using a confidence level of 99% (*t* = 2.6), *d* = 0.065, and a 50% percentage of the population with measurable BPA and nNP levels (*p* = 0.5), the estimated total sample size was 400. Participants in this study were 20–59 years of age, of both sexes, and urban and rural residents. An arbitrary cutoff of 100,000 inhabitants per county was used to distinguish rural from urban areas. Each sample, defined by age (< 50 years or ≥50 years), residence (rural or urban), and sex (male or female), was categorized in eight subpopulation groups (e.g., < 50-year-old rural female).

Because samples were obtained from the NHANES III callback cohort, a nonrepresentative subset of NHANES III samples, the summary statistics are not representative of the U.S. population but serve as reference ranges for the three population breakdowns specified above (i.e., persons < 50 or ≥50 years of age; rural or urban residents; male or female). To improve the extent to which the results represent the U.S. population, we used sample weights. We developed our own weights for demographic groups, not for individual subjects. This approach is different from that used by the National Center for Health Statistics (NCHS) of the Centers for Disease Control and Prevention (CDC). The NCHS assigns a unique weight to each subject based on demographics, geographical data, and oversampling of certain population groups. Because we only had information on age group, sex, and residence (i.e., rural and urban), we could not assign weights to individual subjects, only to the demographic groups. We determined the weights by relating the sample sizes in each of the eight groups to the total numbers of persons in the U.S. population in the same groups defined by sex, residence, and age. From within these eight groups, we randomly selected 394 samples. The institutional review board of the NCHS approved the study.

We analyzed the weighted data using SAS software, version 8.2 (SAS Institute, Cary, NC). Because the base-10 logarithm of the concentrations (log-transformed concentrations) was less skewed than the nontransformed values, we used the log-transformed values in the analyses. We calculated GMs and distribution percentiles for both volume-based (micrograms per liter) and creatinine-corrected concentrations (micrograms per gram creatinine). The GMs were exponentiated results obtained from the means of the log-transformed concentrations. GMs were calculated when the frequency of detection of the analyte was > 60%. We did not use weights to obtain GM or percentile estimates for the various demographic groups because each subject in a demographic group had the same weight.

For exploratory purposes only, we compared BPA and NP levels among subgroups (by age, sex, and place of residence) even though we did not design the study to assure adequate statistical power for this type of hypothesis testing (i.e., the sample size was determined to answer only the question about the percentage of population with measurable urinary BPA and/or NP levels). We used weighted analysis of covariance models to study the effects of residence, sex, age group, and urinary creatinine on the urinary log-transformed concentrations of BPA and NP. The analyses were performed using SAS Proc GENMOD (SAS Institute) to model the log-transformed concentrations (dependent variable) as a function of sex, residence, age group (categorical covariates), and urinary creatinine (continuous covariate used to adjust for urine dilution). The purpose of our model adjustment was not to apply an individual adjustment to BPA and NP concentrations, but rather to enable us to determine whether there are differences in average BPA or NP urinary levels between individuals in the same demographic groups (e.g., men vs. women) after accounting for the differences due to urinary dilution. By adjusting for creatinine, we obtained a comparison that was not influenced by differences in creatinine levels. We also considered all possible two-way interactions between covariates. Type 3 equivalent sums of squares from the model were used to form likelihood ratio tests of model effects and various tests of hypotheses. Statistical significance was set at *p* < 0.05. We dealt with results < LOD by using a multiple imputation method (Lynn 2001) along with the SAS procedure PROC MIANALYZE, which summarizes parameter estimates and incorporates the resulting uncertainty associated with the multiple imputations used to obtain them.

## Results

**Figure 1:**
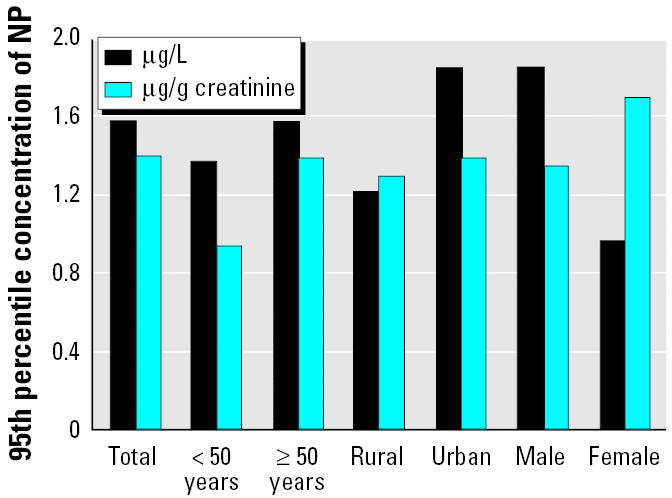
Ninety-fifth percentile concentration (in μg/L and μg/g creatinine) of NP in a human reference population of 371 adults. The frequency of detection of NP was 51%, and the median concentration was < 0.1 μg/L. For each demographic subgroup, the frequency of detection was 53% (< 50 years of age), 45% (≥50 years of age), 51% (rural resident), 51% (urban resident), 55% (male), and 48% (female). The median concentrations of NP were < 0.1 μg/L, except for males (0.17 μg/L), urban residents (0.11 μg/L), and those < 50 years of age (0.11 μg/L).

The distributions of BPA and NP in 394 NHANES III callback cohort urine samples analyzed are shown in Table 1 and Figure 1, respectively. For NP, results were available for 371 samples. Ninety-five percent of the samples measured had detectable concentrations of BPA, suggesting that human exposure to BPA is widespread. NP was detected in 51% of the samples. The lower frequency of NP than BPA detection could be because nNP (the compound we measured) represents a small percentage of the NP present in the NP commercial mixtures, to relative differences in the used amounts of BPA and NP ethoxylates (the environmental precursors of NP), the degree of human exposure, the pathways and routes of exposure, pharmacokinetic factors (i.e., absorption, distribution, metabolism, and elimination), or a combination of all of the above.

Beginning with the initial BPA model described above, we arrived at a final model that included residence, creatinine, and residence-by-creatinine interaction (Table 2). We calculated adjusted GM estimates of BPA for residence groups by using the mean values of the continuous covariate (i.e., creatinine). For BPA, the main effects for sex and age group were not statistically significant, and they were excluded from the final model. In contrast, the residence × creatinine term was statistically significant (*p* = 0.0249), suggesting that the estimated difference in BPA concentrations between urban and rural residents depended upon the urinary creatinine level, and that the relationship between urinary levels of BPA and creatinine is not the same for urban and rural residents. The adjusted GM of BPA for an urban resident (1.21 μg/L) was significantly lower (*p* = 0.0014) than that for a rural resident (1.56 μg/L). These values (Table 2) compared well with the observed values (Table 1). According to the model results, BPA levels for rural residents were expected to be 1.28 [95% confidence interval (CI), 1.05–1.57] times higher than those for urban residents assuming a urinary creatinine concentration in both groups of 12.63 mmol/L. Furthermore, on the basis of model results, every mmol/L increase in urinary creatinine levels was expected to be associated with a 5.8% (95% CI, 0.4–8.1%) increase in BPA levels for rural residents and a9.5% (95% CI, 7.2–11.9) increase for urban residents. These data suggest the possibility of variations in exposure to BPA on the basis of place of residence. However, the nature of these variations is not apparent (maybe due to differences in diet, lifestyle, or exposure to other chemicals) and is difficult to explain with the available demographic data.

For NP, no statistically significant interactions existed between any of the covariates considered, and none of the categorical main effects was statistically significant. Only urinary creatinine was statistically significant (*p* < 0.0001). The slope (β) from the multivariate analysis of the regression of log_10_(NP) versus creatinine was 0.036. On the basis of model results, the NP GM concentration was < LOD (0.1 μg/L), assuming all participants had an average urinary creatinine of 12.63 mmol/L. Furthermore, for every milli-mole per liter increase in urinary creatinine, NP concentration increased by 8.6% (95% CI, 5.6–11.7).

Because of the relatively large percentage of NP results < LOD, we performed a logistic regression analysis to determine whether any of the covariates might be associated with an NP level large enough to be detected. The results of this analysis showed no evidence for an effect of the categorical covariates (i.e., sex, residence, age group) on the likelihood (i.e., probability) of a subject having a measurable NP concentration. For every mmol/L increase in creatinine concentrations, the likelihood of a measurable NP level increased by 9.1% (95% CI, 5.6–12.7), confirming that the likelihood of measurable NP levels is associated with increased urine concentration (using creatinine as an indicator of urine concentration) that also is directly related to increased NP concentrations. We found no significant correlation between the levels of urinary BPA and NP (data not shown). These findings suggest that, as expected, no common source of exposure exists for BPA and NP.

## Discussion

We measured BPA and NP in a group of 394 urine samples from the NHANES III callback cohort (Table 1, Figure 1). NHANES III, conducted from 1988 through 1994 by the NCHS (1994), was designed as a nationally representative survey. However, the environmental component, known as the callback cohort (~ 1,000 adults who agreed that additional blood and urine samples could be taken), was not. Although each demographic group had some representation in the call-back cohort, no rigorous sample design and no sample weights were used in analyzing the resulting data. Furthermore, for this study, we calculated the total sample size (~ 400) to assure that we could estimate the true proportion of the population with measurable BPA and/or NP urinary concentrations within 6.5 percentage points with 99% confidence. It is likely that this sample size was too small to detect significant differences between the various subgroups defined by age, residence, and sex. Even though our population does not represent the composition of the general U.S. population and the sample size is relatively small, our reported values are from a diverse adult U.S. population, a larger and broader population base than those in previous studies (Arakawa et al. 2004; Matsumoto et al. 2003; Ouchi and Watanabe 2002; Yang et al. 2003).

The median concentrations of BPA in the NHANES III samples analyzed were similar to the concentrations found in a group of 48 Japanese adults (Ouchi and Watanabe 2002), but the GM concentration of BPA was about seven times lower than in a group of 73 adult Koreans (Yang et al. 2003). These data suggest that differences in the exposure to BPA may exist geographically. However, because of the relatively small sample size of these studies, the data should be interpreted cautiously. The range of NP concentrations in the NHANES III samples compared well with previous data (Inoue et al. 2003; Kawaguchi et al. 2004).

The relatively low NP concentrations and lower frequency of detection compared with that of BPA may result, at least in part, from alternative metabolic pathways. Orally administered BPA is rapidly metabolized to its monoglucuronide in rats (Pottenger et al. 2000) and humans (Volkel et al. 2002) and excreted in the urine. In six adult volunteers administered orally with D_16_-BPA (5 mg), D_16_-BPA was rapidly absorbed from the gastrointestinal tract. The urinary excretion and elimination from blood of D_16_-BPA glucuronide were very similar, with terminal half-lives of 5.4 hr and 5.3 hr, respectively. Furthermore, the applied dose of D_16_-BPA was completely recovered in urine as D_16_-BPA glucuronide approximately 24 hr after administration (Volkel et al. 2002). In turn, after oral application of ^13^C_6_-NP (5 mg) to a human volunteer, Muller et al. (1998) showed that only 10% of the dose was excreted in the urine as NP within 8 hr and suggested the occurrence of additional metabolites, which could not be identified with their method. Other studies have demonstrated that NP undergoes metabolism in fish (Coldham et al. 1998; Thibaut et al. 1998, 1999) and rats (Doerge et al. 2002; Green et al. 2003; Zalko et al. 2003) resulting both in side chain- and ring-hydroxylated NP metabolites. In Wistar rats, a major percentage (~ 75%) of ring-2,6–^3^H–nNP, administered by gavage, or oral administered ^14^C_6_-nNP was excreted in urine in the form of free, glucuronidated, or sulfated oxidative metabolites, primarily C1 and C3 side-chain oxidative metabolites resulting from ω- or β-oxidation of the NP C9 side chain (Zalko et al. 2003). Several metabolites were characterized by negative ion electrospray-ion trap MS, namely, *p*-hydroxybenzoic acid, 3-(4-hydroxyphenyl)-2-propionic acid, 3-(4-hydroxyphenyl)-2-propenoic acid, and a ring-hydroxylated 3-(4-hydroxyphenyl)-2-propionic acid. No nNP was detected in urine (Zalko et al. 2003). In rainbow trout, after oral ingestion of ring-2,6–^3^H–nNP, the major urinary metabolites were tentatively identified as oxidative metabolites (Thibaut et al. 1999). In another study, the excretion in urine of unchanged ^14^C_6_-nNP in CD rats after oral administration of the radiolabeled NP was estimated to be < 5% of the dose (Green et al. 2003). In Sprague-Dawley rats, after oral administration of NP, containing 95% of branched side-chain NP isomers, considerable amounts of aromatic ring hydroxylated glucuronides were detected in serum and liver based on MS fragmentation properties (Doerge et al. 2002; Green et al. 2003; Zalko et al. 2003).

If the oxidative metabolism of NP also prevails in humans, the use of NP as the sole urinary biomarker for comparing relative exposures of NP to other environmental phenols (e.g., BPA) in a given study may be misleading because the metabolism of NP is more complex and results in more metabolites, thus decreasing the relative amounts of NP in the urine. Although ingested BPA is completely recovered in urine as BPA glucuronide within approximately 24 hr after exposure (Volkel et al. 2002), only 10% of the ingested NP is excreted in the urine as NP or conjugated NP (e.g., glucuronide) (Muller et al. 1998). Furthermore, given the method of manufacturing NP, little of the linear chain NP (i.e., nNP) is produced (EC 2002a). Therefore, nNP, the compound we actually measured in this study, represented only a small percentage of the NP present in the NP commercial mixtures. Unfortunately, the lack of authentic standards and isotope-labeled standards of branched NPs precluded their quantification (Kuklenyik et al. 2003). Studies are ongoing in our laboratory to determine whether the NP oxidative metabolites tentatively identified in animal studies, including the metabolites of branched alkyl chain NPs, can be used to assess exposure to NP in humans.

In summary, despite the sample population’s relatively small size and its nonrepresentativeness of the U.S. population (although we used sample weights to improve the extent to which the results represent the U.S. population), this study provides the first reference range of human internal dose levels of BPA and NP. The higher frequency of detection of BPA than of NP in urine suggests that human exposure to BPA is more prevalent. However, because an oxidative metabolism pathway for NP may be relevant in humans and nNP—the NP isomer measured in this study—represents a small percentage of the NP used in commercial mixtures, exposure to NP may be underestimated. Additional research to establish the relevance of oxidative metabolism of NP in humans and to identify the urinary metabolites of NP commercial mixtures is warranted. Furthermore, the frequent human exposure to these compounds highlights the need for future studies to measure BPA and NP in a nationally representative sample of the U.S. population.

## Figures and Tables

**Table 1:** GM and selected percentiles of BPA concentrations [μg/L (μg/g creatinine)] in urine.

		Percentile						
	GM	10th	25th	50th	75th	90th	95th	No. (%)*^a^*
Total	1.33 (1.36)	0.22 (0.23)	0.58 (0.70)	1.28 (1.32)	2.46 (2.58)	4.10 (3.88)	5.18 (7.95)	394 (95)
Age group (years)
< 50	1.43 (1.34)	0.44 (0.44)	0.84 (0.74)	1.87 (1.53)	3.13 (2.34)	5.15 (3.77)	7.51 (6.64)	317 (95)
≥50	0.99 (1.44)	0.18 (0.15)	0.51 (0.65)	1.23 (1.29)	2.32 (2.60)	4.00 (3.88)	4.83 (7.95)	77 (94)
Place of residence
Rural	1.44 (1.60)	0.38 (0.36)	0.69 (0.73)	1.28 (1.37)	2.35 (2.58)	3.79 (3.83)	4.76 (9.06)	153 (99)
Urban	1.27 (1.23)	0.18 (< LOD)	0.41 (0.53)	1.27 (1.20)	2.58 (2.60)	4.42 (3.88)	6.26 (7.95)	241 (92)
Sex
Male	1.63 (1.35)	0.40 (0.15)	0.71 (0.57)	1.30 (1.20)	2.36 (1.91)	4.42 (3.05)	8.02 (7.95)	184 (96)
Female	1.12 (1.35)	0.17 (0.36)	0.49 (0.72)	1.27 (1.77)	2.46 (2.95)	3.68 (4.49)	4.83 (9.06)	210 (94)

LOD is 0.1 μg/L.

a Number of subjects (percentage of detection).

**Table 2:** Multivariate model*^a^* considering the relation of measured BPA*^b^* to independent predictor variables.

Independent variable	Specification	No.	GM*^c^*	*p*-Value
Residence	Rural	153	1.56	0.0014
	Urban	241	1.21	
Creatinine	Continuous	394	0.039*^d^*	< 0.0001
Residence × creatinine	Rural	153	−0.015*^d^*	0.0249
	Urban	241		

a Each variable was adjusted for the others in the model; *r*^2^ for the model is 0.26.

b The dependent variable is log_10_(BPA) for model calculations (μg/L).

c Model-calculated GM.

d Values shown are β[i.e., slope from the multivariate analysis of the regression of log_10_(BPA) versus the continuous independent variable].

## References

[b1-ehp0113-000391] Arakawa C, Fujimaki K, Yoshinaga J, Imai H, Serizawa S, Shiraishi H (2004). Daily urinary excretion of bisphenol A. Environ Health Prevent Med.

[b2-ehp0113-000391] Arenholt-Bindslev D, Breinholt V, Preiss A, Schmalz G (1999). Time-related bisphenol-A content and estrogenic activity in saliva samples collected in relation to placement of fissure sealants. Clin Oral Investig.

[b3-ehp0113-000391] Coldham NG, Sivapathasundaram S, Dave M, Ashfield LA, Pottinger TG, Goodall C (1998). Biotransformation, tissue distribution, and persistence of 4-nonylphenol residues in juvenile rainbow trout (*Oncorhynchus mykiss*). Drug Metabol Dispos.

[b4-ehp0113-000391] Doerge DR, Twaddle NC, Churchwell MI, Chang HC, Newbold RR, Delclos KB (2002). Mass spectrometric determination of *p*-nonylphenol metabolism and disposition following oral administration to Sprague-Dawley rats. Reprod Toxicol.

[b5-ehp0113-000391] EC 2002a. 4-Nonylphenol (Branched) and Nonylphenol. Risk Assessment Report and Summary Vol 10. Luxembourg: European Commission. Available: http://ecb.jrc.it/DOCUMENTS/Existing-Chemicals/RISK_ASSESSMENT/REPORT/4-nonylphenol_nonylphenolreport017.pdf [accessed 15 December 2004].

[b6-ehp0113-000391] EC 2002b. Opinion of the Scientific Committee on Food on Bisphenol A. Brussels, Belgium:European Commission. Available: http://europa.eu.int/comm/food/fs/sc/scf/out128_en.pdf [accessed 26 May 2004].

[b7-ehp0113-000391] Green T, Swain C, Van Miller JP, Joiner RL (2003). Absorption, bioavailability, and metabolism of para-nonylphenol in the rat. Regul Toxicol Pharmacol.

[b8-ehp0113-000391] Guenther K, Heinke V, Thiele B, Kleist E, Prast H, Raecker T (2002). Endocrine disrupting nonylphenols are ubiquitous in food. Environ Sci Technol.

[b9-ehp0113-000391] Hanaoka T, Kawamura N, Hara K, Tsugane S (2002). Urinary bisphenol A and plasma hormone concentrations in male workers exposed to bisphenol A diglycidyl ether and mixed organic solvents. Occup Environ Med.

[b10-ehp0113-000391] Hassold T, Hunt P (2001). To ERR (meiotically) is human: the genesis of human aneuploidy. Nat Rev Genet.

[b11-ehp0113-000391] Hemmer MJ, Hemmer BL, Bowman CJ, Kroll KJ, Folmar LC, Marcovich D (2001). Effects of *p*-nonylphenol, methoxychlor, and endosulfan on vitellogenin induction and expression in sheepshead minnow (*Cyprinodon varie-gatus*). Environ Toxicol Chem.

[b12-ehp0113-000391] Howdeshell KL, Hotchkiss AK, Thayer KA, Vandenbergh JG, vom Saal FS (1999). Environmental toxins—exposure to bisphenol A advances puberty. Nature.

[b13-ehp0113-000391] Howe SR, Borodinsky L, Lyon RS (1998). Potential exposure to bisphenol A from food contact use of epoxy coated cans. J Coat Technol.

[b14-ehp0113-000391] Hunt PA, Koehler KE, Susiarjo M, Hodges CA, Ilagan A, Voigt RC (2003). Bisphenol A exposure causes meiotic aneuploidy in the female mouse. Curr Biol.

[b15-ehp0113-000391] Inoue K, Kawaguchi M, Okada F, Takai N, Yoshimura Y, Horie M (2003). Measurement of 4-nonylphenol and 4-*tert*-octylphenol in human urine by column-switching liquid chromatography-mass spectrometry. Anal Chim Acta.

[b16-ehp0113-000391] Jackson S (1966). Creatinine in urine as an index of urinary excretion rate. Health Phys.

[b17-ehp0113-000391] Kawaguchi M, Inoue K, Sakui N, Ito R, Izumi S, Makino T (2004). Stir bar sorptive extraction and thermal desorption-gas chromatography-mass spectrometry for the measurement of 4-nonylphenol and 4-*tert*-octylphenol in human biological samples. J Chromatogr B Analyt Technol Biomed Life Sci.

[b18-ehp0113-000391] Kim HS, Shin JH, Moon HJ, Kang IH, Kim TS, Kim IY (2002). Comparative estrogenic effects of *p*-nonylphenol by 3-day uterotrophic assay and female pubertal onset assay. Reprod Toxicol.

[b19-ehp0113-000391] Kubo K, Arai O, Omura M, Watanabe R, Ogata R, Aou S (2003). Low dose effects of bisphenol A on sexual differentiation of the brain and behavior in rats. Neurosci Res.

[b20-ehp0113-000391] Kuklenyik Z, Ekong J, Cutchins CD, Needham LL, Calafat AM (2003). Simultaneous measurement of urinary bisphenol A and alkylphenols by automated solid-phase extractive derivatization gas chromatography/mass spectrometry. Anal Chem.

[b21-ehp0113-000391] Kwack SJ, Kwon O, Kim HS, Kim SS, Kim SH, Sohn KH (2002). Comparative evaluation of alkylphenolic compounds on estrogenic activity in vitro and in vivo. J Toxicol Environ Health Part A.

[b22-ehp0113-000391] Laws SC, Carey SA, Ferrell JM, Bodman GJ, Cooper RL (2000). Estrogenic activity of octylphenol, nonylphenol, bisphenol A and methoxychlor in rats. Toxicol Sci.

[b23-ehp0113-000391] Lynn HS (2001). Maximum likelihood inference for left-censored HIV RNA data. Statist Med.

[b24-ehp0113-000391] Maguire RJ (1999). Review of the persistence of nonylphenol and nonylphenol ethoxylates in aquatic environments. Water Qual Res J Canada.

[b25-ehp0113-000391] Markey CM, Luque EH, de Toro MM, Sonnenschein C, Soto AM (2001). In utero exposure to bisphenol A alters the development and tissue organization of the mouse mammary gland. Biol Reprod.

[b26-ehp0113-000391] Matsumoto A, Kunugita N, Kitagawa K, Isse T, Oyama T, Foureman GL (2003). Bisphenol A levels in human urine. Environ Health Perspect.

[b27-ehp0113-000391] Montgomery-Brown J, Reinhard M (2003). Occurrence and behavior of alkylphenol polyethoxylates in the environment. Environ Engineer Sci.

[b28-ehp0113-000391] Muller S, Schmid P, Schlatter C (1998). Pharmacokinetic behavior of 4-nonylphenol in humans. Environ Toxicol Pharmacol.

[b29-ehp0113-000391] Nagel SC, vom Saal FS, Thayer KA, Dhar MG, Boechler M, Welshons WV (1997). Relative binding affinity serum modified access (RBA-SMA) assay predicts the relative *in vivo* bioactivity of the xenoestrogens bisphenol A and octylphenol. Environ Health Perspect.

[b30-ehp0113-000391] NCHS (1994). Plan and operation of the Third National Health and Nutrition Examination Survey, 1988–94. Vital Health Stat 1.

[b31-ehp0113-000391] Ouchi K, Watanabe S (2002). Measurement of bisphenol A in human urine using liquid chromatography with multi-channel coulometric electrochemical detection. J Chromatogr B Analyt Technol Biomed Life Sci.

[b32-ehp0113-000391] Peavy JV 1996. Surveys and sampling. In: Field Epidemiology (Gregg MB, ed). New York:Oxford University Press, 152–163.

[b33-ehp0113-000391] Pottenger LH, Domoradzki JY, Markham DA, Hansen SC, Cagen SZ, Waechter JM (2000). The relative bioavailability and metabolism of bisphenol A in rats is dependent upon the route of administration. Toxicol Sci.

[b34-ehp0113-000391] Sajiki J, Yonekubo J (2003). Leaching of bisphenol A (BPA) to sea-water from polycarbonate plastic and its degradation by reactive oxygen species. Chemosphere.

[b35-ehp0113-000391] Schonfelder G, Wittfoht W, Hopp H, Talsness CE, Paul M, Chahoud I (2002). Parent bisphenol A accumulation in the human maternal–fetal–placental unit. Environ Health Perspect.

[b36-ehp0113-000391] Staples CA, Dorn PB, Klecka GM, O’Block ST, Harris LR (1998). A review of the environmental fate, effects, and exposures of bisphenol A. Chemosphere.

[b37-ehp0113-000391] Thibaut R, Debrauwer L, Rao D, Cravedi JP (1998). Disposition and metabolism of [H-3]-4-n-nonylphenol in rainbow trout. Mar Environ Res.

[b38-ehp0113-000391] Thibaut R, Debrauwer L, Rao D, Cravedi JP (1999). Urinary metabolites of 4-n-nonylphenol in rainbow trout (*Oncorhynchus mykiss*). Sci Tot Environ.

[b39-ehp0113-000391] U.S. EPA 2003. Ambient Aquatic Life Water Quality Criteria for Nonylphenol—Draft. Washington, DC:U.S. Environmental Protection Agency. Available: http://www.epa.gov/waterscience/criteria/nonylphenol/draft-nonylphenol.pdf [accessed 23 May 2004].

[b40-ehp0113-000391] Volkel W, Colnot T, Csanady GA, Filser JG, Dekant W (2002). etabolism and kinetics of bisphenol A in humans at low doses following oral administration. Chem Res Toxicol.

[b41-ehp0113-000391] vom Saal FS, Cooke PS, Buchanan DL, Palanza P, Thayer KA, Nagel SC (1998). A physiologically based approach to the study of bisphenol A and other estrogenic chemicals on the size of reproductive organs, daily sperm production, and behavior. Toxicol Ind Health.

[b42-ehp0113-000391] Welshons WV, Nagel SC, vom Saal FS (1998). The importance of protocol design and data reporting to research on endocrine disruption: response [Letter]. Environ Health Perspect.

[b43-ehp0113-000391] White R, Jobling S, Hoare SA, Sumpter JP, Parker MG (1994). nvironmentally persistent alkylphenolic compounds are estrogenic. Endocrinology.

[b44-ehp0113-000391] Yang MH, Kim SY, Lee SM, Chang SS, Kawamoto T, Jang JY (2003). Biological monitoring of bisphenol A in a Korean population. Arch Environ Contam Toxicol.

[b45-ehp0113-000391] Ying GG, Williams B, Kookana R (2002). Environmental fate of alkylphenols and alkylphenol ethoxylates. A review. nviron Int.

[b46-ehp0113-000391] Zalko D, Costagliola R, Dorio C, Rathahao E, Cravedi JP (2003). In vivo metabolic fate of the xeno-estrogen 4-*n*-nonylphenol in Wistar rats. Drug Metabol Dispos.

